# Recent advances in uncovering the mechanisms contributing to BIRD-2-induced cell death in B-cell cancer cells

**DOI:** 10.1038/s41419-018-1297-z

**Published:** 2019-01-17

**Authors:** Martijn Kerkhofs, Tamara Vervloessem, Mart Bittremieux, Geert Bultynck

**Affiliations:** KU Leuven, Laboratory of Molecular and Cellular Signaling, Department of Cellular and Molecular Medicine and Leuven Kanker Instituut (LKI), Campus Gasthuisberg ON-I Bus 802, Leuven, Belgium

A common observation in hematological cancer cells, including follicular lymphoma, diffuse large B-cell lymphoma (DLBCL) and chronic lymphocytic leukemia (CLL), is the upregulation of the anti-apoptotic B-cell lymphoma 2 (Bcl-2) protein, the founding member of the Bcl-2-protein family^1^. Bcl-2 overexpression enables cancer cell survival despite pro-apoptotic challenges related to oncogenic stress such as genomic aberrations^1^. Bcl-2 provides this protection by acting at the mitochondrial outer membrane, scaffolding pro-apoptotic Bcl-2-family members such as Bax and Bak (multi-domain executioners of mitochondrial outer membrane permeabilization), and Bim (a BH3-only protein activating Bax and Bak) via its hydrophobic cleft, that is formed by the B-cell homology (BH)1, -2, and -3 domains^1^. In cancer cells, pro-apoptotic factors (such as Bim) are often upregulated, establishing a dependency on anti-apoptotic Bcl-2 to prevent apoptosis. This dependency is exploited by BH3-mimetic anticancer agents, such as ABT-737 and ABT-199 (venetoclax), which antagonize Bcl-2 at the level of the hydrophobic cleft^1^. Recently, venetoclax has been approved by the Food and Drug Administration (FDA) for the treatment of patients with relapsed CLL^2^.

However, it has become clear that Bcl-2 overexpression can also protect cells against apoptosis through means other than its canonical anti-apoptotic function^3^. Indeed, work from several labs indicated that Bcl-2 is present at the endoplasmic reticulum (ER) Ca^2+^ stores, where it diminishes Ca^2+^ efflux from the ER^4^. Although different mechanisms have been proposed, it is clear that Bcl-2, via its BH4 domain, can directly bind IP_3_ receptors (IP_3_Rs)—intracellular Ca^2+^-release channels—and limit their Ca^2+^-flux properties, thereby preventing cell death driven by Ca^2+^ overload^5^.

Bcl-2-IP_3_R disrupter-2 (BIRD-2), a cell-permeable peptide tool that targets Bcl-2’s BH4 domain has been developed by fusing the TAT sequence to a stretch of 20 amino acids representing the Bcl-2-binding site present in the central, modulatory region of the IP_3_R^6,7^. This peptide is able to disrupt the interaction between the IP_3_R and Bcl-2^8^. BIRD-2 provoked spontaneous IP_3_R-mediated Ca^2+^ signaling and cell death in several Bcl-2-dependent cancer cell models, including CLL, multiple myeloma and follicular lymphoma^9^, small cell lung cancer, and DLBCL^7^. Interestingly, in DLBCL at least, we discovered a negative correlation between the sensitivity towards venetoclax and BIRD-2^10^. Therefore, we may speculate that a cancer cell needs to choose to deploy Bcl-2 for its canonical role at the mitochondria, preventing Bax/Bak activity, or an alternative function at the ER, inhibiting IP_3_R activity. The former depends on Bcl-2’s hydrophobic cleft, whereas its BH4 domain is involved in the latter.

Recent work from our lab has shed more light on the mechanism of action of BIRD-2. A paper by Bittremieux et al. highlights the importance of intra- and extracellular Ca^2+^ for BIRD-2 to work^11^. We initially hypothesized that store-operated Ca^2+^ entry (SOCE) is an important process in BIRD-2-induced cell death. After all, BIRD-2 promotes Ca^2+^ release from the ER, which would be refilled upon depletion by SOCE. During Ca^2+^ depletion, the luminal ER Ca^2+^ sensor STIM1, interacts with ORAI, a plasma membrane resident Ca^2+^-influx channel. This interaction results in the activation of ORAI and Ca^2+^ influx, refilling the ER. However, Bittremieux et al. showed that SOCE is not necessary for BIRD-2-induced cell death. They did this by using several well-characterized pharmacological tools, including DPB162-AE, YM-58483, and GSK-7975A. All compounds were shown to inhibit SOCE, but, interestingly, only DPB162-AE could reduce BIRD-2-induced cell death. This discrepancy was explained by DPB162-AE’s effect on ER Ca^2+^ store filling, since treatment with thapsigargin and cyclopiazonic acid, two other molecules reducing the ER Ca^2+^ store but without effect on SOCE, too, could protect against BIRD-2-induced cell death. These experiments confirm and highlight the importance of ER Ca^2+^ in BIRD-2’s working mechanism. The case against the involvement of SOCE in BIRD-2-mediated cell death was strengthened by a knock-down of STIM1. Cell death experiments comparing the knock-down and the wild-type showed no significant difference between the two conditions^11^. Caution with the interpretation of these results is warranted, since both the pharmacological and genetic approaches may not have completely annihilated SOCE and thus remnant SOCE could have been sufficient for BIRD-2-induced cell death.

Although SOCE was excluded as a major factor in the cell death mechanism underlying BIRD-2, there was an indication that extracellular Ca^2+^ is important for proper cell death induction by BIRD-2^11^. Experiments performed with ethylene glycol-bis(β-aminoethyl ether)-N,N,N′,N′-tetraacetic acid (EGTA) in the extracellular medium showed that the intracellular Ca^2+^ signal, elicited by BIRD-2, is not present when Ca^2+^ is chelated in the extracellular environment. This implies that extracellular Ca^2+^ is involved in killing the cells with BIRD-2. However, the molecular identity of the pathway mediating Ca^2+^ influx from the extracellular medium remains elusive and requires further investigation^11^.

Independently from this, our lab has also identified other factors that contribute to the sensitivity of DLBCL cancer cells towards BIRD-2 exposure (Fig. 1). A first factor is the expression of particular IP_3_R isoforms^12^. We found that cells displaying high IP_3_R2 subtype expression are most sensitive towards BIRD-2. It is hypothesized that these cells are more sensitive to disinhibition of the IP_3_R due to Bcl-2 removal from the channel, because the IP_3_R2 has the highest affinity for its ligand IP_3_^12^. A second factor that contributes to BIRD-2 sensitivity is constitutive IP_3_ signaling^13^. B-cell cancers are often characterized by chronic or tonic B-cell receptor (BCR) activity. Importantly, phospholipase γ2, an enzyme producing IP_3_ and diacyl glycerol from phosphatidylinositol 4,5-bisphosphate (PIP_2_) present in the cell membrane, acts downstream of this hyperactive BCR, thus providing a constant source of IP_3_ that helps to promote cell survival and growth^14^. Treatment of DLBCL and primary CLL cells with a chemical inhibitor of phospholipase C suppressed the ability of BIRD-2 to provoke cell death. At least in DLBCL cell lines, these pharmacological experiments were independently validated by the overexpression of an IP_3_ sponge that buffers free IP_3_, thereby dampening BIRD-2-induced cell death. So, although these tumor cells use constitutive IP_3_ signaling as a pro-survival mechanism, this signaling system can be converted into a pro-death signal by BIRD-2^13^. Now, further research is needed to examine whether BIRD-2 can also kill other primary cancer cells besides the ones derived from CLL patients and whether BIRD-2 sensitivity is dependent on IP_3_R2 expression and IP_3_ signaling in these primary cells.

**Fig. 1:**
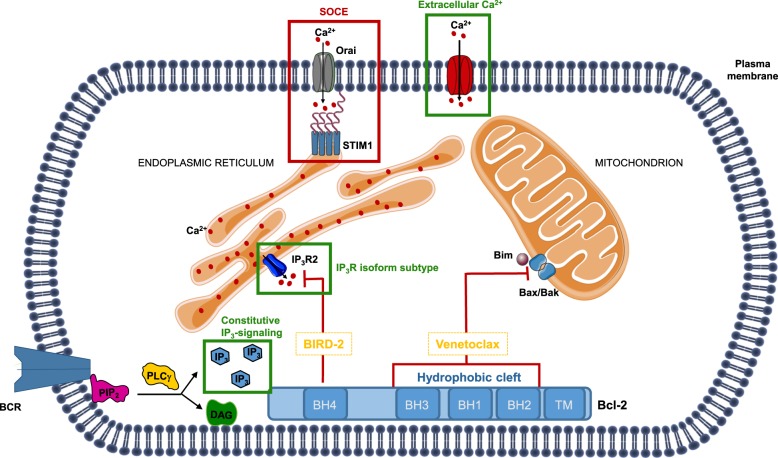
Antagonizing B-cell lymphoma 2 (Bcl-2) to induce cell death in B-cell cancer cells. Two functional domains, the hydrophobic cleft and the BH4 domain, are important for Bcl-2’s anti-apoptotic function. The hydrophobic cleft of Bcl-2 prevents apoptosis by scaffolding and neutralizing several pro-apoptotic Bcl-2 family members, including Bax/Bak and BH3-only proteins such as Bim, at the mitochondrial outer membranes. The hydrophobic cleft of Bcl-2 can be targeted by so-called BH3 mimetics, including the recently FDA-approved small molecule venetoclax/ABT-199, provoking cell death in Bcl-2-dependent cancer cells. The BH4 domain suppresses apoptosis by binding and inhibiting the IP_3_R, intracellular Ca^2+^-release channels present in the endoplasmic reticulum (ER). A decoy peptide, the Bcl-2 IP_3_R disruptor-2 (BIRD-2), can target Bcl-2’s BH4 domain, thereby disrupting Bcl-2/IP_3_R complexes and provoking Ca^2+^-driven apoptosis in Bcl-2-dependent cancer cells. The IP_3_R isoform subtype (IP_3_R2), constitutive IP_3_ signaling and extracellular Ca^2+^ are critical factors that contribute to the sensitivity of Bcl-2-dependent cancer cells towards BIRD-2 (indicated in green), while store-operated Ca^2+^ entry likely may not be involved (indicated in red)

Finally, BIRD-2 can be used to eradicate cancer cells, even when it is not directly killing the cells itself. In ovarian cancer cells, Bcl-2 has been implicated in cisplatin resistance. Recent work by Xie et al. shows that BIRD-2 can overcome cisplatin resistance, thereby re-sensitizing ovarian cancer cells towards cisplatin^15^. At the mechanistic level, BIRD-2 augmented cisplatin-induced Ca^2+^ release and cell death without causing cell death by itself in these cells. These findings would advocate for opportunities to apply BIRD-2 as an adjuvant for other anticancer treatments that impinge on Ca^2+^ signaling^15^.

## References

[1] Montero J (2018). Cell Death Differ..

[2] Green DR (2016). Cell.

[3] Akl H (2014). Biochim. Biophys. Acta Mol. Cell Res..

[4] Vervliet T (2016). Oncogene.

[5] Rong YP (2009). Proc. Natl. Acad. Sci. USA.

[6] Distelhorst, C. W. Creating a New Cancer Therapeutic Agent by Targeting Bcl-2-IP3R Interaction. *Cold Spring Harb. Perspect. Biol*. (2019) (in press). 10.1101/cshperspect.a035196 PMC6719601 31110129

[7] Distelhorst CW (2018). Biochim. Biophys. Acta Mol. Cell Res..

[8] Zhong F (2011). Blood.

[9] Lavik AR (2015). Oncotarget.

[10] Vervloessem T (2017). Oncotarget.

[11] Bittremieux M (2018). Cell Death Discov..

[12] Akl H (2013). Cell Death Dis..

[13] Bittremieux, M. et al. Constitutive IP_3_ signaling underlies the sensitivity of B-cell cancers to the Bcl-2/IP_3_ receptor disruptor BIRD-2. *Cell Death Differ*. 10.1038/s41418-018-0142-3 (2018) 10.1038/s41418-018-0142-3 PMC6370760 29899382

[14] Fowler N (2013). Hematol. Am. Soc. Hematol. Educ. Program.

[15] Xie Q (2018). Int J. Mol. Med..

